# Effect of AIDS-defining events at initiation of antiretroviral therapy on long-term mortality of HIV/AIDS patients in Southwestern China: a retrospective cohort study

**DOI:** 10.1186/s12981-020-00300-4

**Published:** 2020-07-17

**Authors:** Yunxuan Huang, Oulu Zhou, Zhigang Zheng, Yuexiang Xu, Yi Shao, Chunwei Qin, Fengxiang Qin, Jingzhen Lai, Huifang Liu, Rongfeng Chen, Li Ye, Hao Liang, Xionglin Qin, Junjun Jiang

**Affiliations:** 1 Guigang Center for Disease Control and Prevention, Guigang, 537100 Guangxi China; 2 Life Sciences Institute, Guangxi Medical University, Nanning, 530021 Guangxi China; 3 Guangxi Key Laboratory of AIDS Prevention and Treatment, Guangxi Medical University, Nanning, 530021 Guangxi China; 4 Guigang Maternal and Child Health Hospital, Guigang, 537100 Guangxi China

**Keywords:** HIV, AIDS, Antiretroviral therapy, AIDS-defining events, Mortality

## Abstract

**Objective:**

To evaluate the impact of AIDS-defining events (ADE) on long-term mortality of HIV positive individuals on antiretroviral therapy (ART), a retrospective HIV/AIDS treatment cohort study performed in Southwestern China.

**Methods:**

The retrospective cohort was conducted among 6757 HIV/AIDS patients on ART (2NRTIs + 1NNRTI, 2NRTIs + 1PI and Single or two drugs) recruited in Guigang city, Guangxi, China, from January 2004 to December 2018. Participants were divided into ADE and non-ADE groups, and were followed-up every six months to observe treatment outcomes. Comparison of mortality between groups was performed using the log-rank test and Kaplan–Meier analysis. Cox proportional hazard regression was used to explore the risk factors of mortality. 1:1 propensity score matching (PSM) was used to balance confounding factors and adjust the mortality risk.

**Results:**

Of 6757 participants with 29,096.06 person-years of follow-up, 16.86% (1139/6757) belonged to ADE group while the others (83.14%) belonged to the non-ADE group. The most common cause of death by ADE was disseminated mycosis (31.65%), followed by recurrent severe bacterial pneumonia (28.48%), herpes zoster (17.72%), and extra-pulmonary tuberculosis (8.86%). The mortality of the ADE group was significantly higher than that of the non-ADE group [3.45/100 person-years (95% CI 2.92–3.97) vs. 2.34/100 person-years (95% CI 2.15–2.52), *P*<0.001]. The death risk of the ADE group was also higher than that of the non- ADE group [adjusted hazard ratio (aHR) = 1.291, 95% CI 1.061–1.571, *P *= 0.011], which was confirmed by PSM analysis (aHR = 1.581, 95% CI 1.192–2.099, *P *= 0.002). Cox analysis indicated that ADE, older age, male gender, previous non-use of cotrimoxazole, advanced WHO clinical stage, and low baseline CD4^+^ cell count were the risk factors for death.

**Conclusions:**

Even on ART, the mortality risk of HIV positive individuals with ADE was higher than those without ADE. Active testing, earlier diagnosis, and timely therapy with ART may reduce the death risk of ADE.

## Introduction

Since the first case of the human immunodeficiency virus (HIV) infection was reported in 1981, 36.9 million people have been infected with HIV; the worldwide mortality rate of HIV/AIDS patients was 23.8% in 2017 [1]. As of August 31, 2018, 841,478 living HIV/AIDS patients and 259,200 deaths had been registered in China, with mortality rate of 23.55% [2, 3]. Guangxi, a province in Southwestern China, has the second highest number of reported HIV cases in the country, with 703,000 survivals and 40,500 deaths by 2017. The mortality rate (34.3%) was also higher than the national average mortality rate (23.8%) during the same period [4]. In China, the National Free Antiretroviral Treatment Program (NFATP) began in 2002 and was scaled up in 2003. The “Treat for All” policy was implemented in 2016, and HIV positive individuals would be treated after diagnosis of HIV regardless of their CD4^+^ cell count [5–7]. However, research showed that 45.1% patients were in the advanced stages of AIDS when they were diagnosed [8], which might be the main reason for the high mortality rate in HIV positive individuals, even when they are on ART.

ART can significantly improve the prognosis of HIV infected people, and reduce the spread of HIV, which is more conducive to improving the prognosis and quality of life of patients [9]. At present, anti HIV drugs can be divided into six categories: nucleoside reverse transcriptase inhibitors (NRTIs), nonnucleoside reverse transcriptase inhibitors (NNRTIs), protease inhibitors (PIs), integrase inhibitors (INIs), fusion inhibitors and entry inhibitors. Then, the most widely used drugs are the first three categories in China. NRTIs mainly contains zidovudine (AZT), stavudine (D4T), lamivudine (3TC), tenofovir (TDF) and abacavir (ABC) et al.; NNRTIs mainly contains nevirapine (NVP), delavirdine, efavirenz (EFV) and etravirine et al.; and PIs mainly contains lopinavir (LPV), saquinavir, ritonavir and indinavir. In this study, the patients mainly used three kinds of antiviral treatment schemes: 2NRTIs + 1NNRTI, 2NRTIs + 1PI and single or the combination of two drugs.

By 2017, 542,000 people living with HIV/AIDS (PLHIV) had received ART in China [2]. Research showed that ART also can effectively reduce mortality, delay the progression of the disease, prolong the development of non-AIDS-defining events (non-ADE) to ADE, reduce complications, and prolong lifespan [10]. However, ART-treated patients still face a higher mortality risk and lower quality of life than the general population [11–14]. Many studies have shown that a low CD4^+^ cell count, older age, male gender, clinical stage III/IV disease, low body mass index (BMI), and signs/symptoms of AIDS are independent factors related to death among patients on ART [4, 15–19], indicating that patients with ADE may have higher mortality. A study in Italy suggested that patients with ADE should be treated within 30 days after ADE diagnosis. However, only 43% of ADE patients in Italy received treatment [20]. A recent study also found that the presence of AIDS-defining disease at HIV diagnosis was one of the factors related to high mortality [21].

So far, a number of studies have assessed in the relationship between ART and ADE, focusing on the relationships between ART [22–24], quality of life [25], cytomegalovirus infection [26], and the effect of the timing of ART on survival [27]. However, most studies in the field have explored the impact of ART on ADE. A retrospective study showed that 57% of patients with advanced HIV disease had opportunistic infections, and the majority were diagnosed when they had developed ADE [28]. Late diagnosis and delayed treatment of HIV infection contribute to the development of opportunistic infections, such as pneumocystis pneumonia, Talaromyces marneffei infection, etc. In Guangxi, a recent retrospective study revealed that 70.2% of newly diagnosed HIV cases from 2012 to 2016 had a late presentation, and 45.1% had advanced HIV disease [29]. Most patients with late presentation, advanced HIV disease, or ADE have relatively low CD4^+^ cell count or develop AIDS symptoms.

Comprehensively, we speculate that the high mortality rate of HIV/AIDS patients in Guangxi, China may be due to the high proportion of ADE in the ART population. Therefore, in this study, we aimed to evaluate the effect of ADE on the mortality rate of HIV/AIDS patients who were on ART, providing a basic understanding of the relationship between ART and ADE from a perspective different from those of previous studies.

## Methods

### Study site and population

The HIV/AIDS treatment cohort in Guigang city, Guangxi, China was constructed from the treatment system starting in 2004. It was regarded as one of the longest observational treatment cohorts in Guangxi, since the treatment system was initiated simultaneously with the NFATP in China. We retrospectively extracted demographic information and data for ART-received patients who registered in the system between January 2004 and December 2018. The inclusion criteria for participants were as follows: (1) HIV-positive; (2) age at least 18 years; (3) received ART for the first time; (4) the disease category was recorded within 3 months of initiating antiviral treatment.

### Study design

A retrospective cohort study was conducted among HIV/AIDS patients from NFATP at Guigang Center for Disease Control and Prevention, HIV/AIDS individuals who receiving ART from January 2004 to December 2018 were recruited for analysis. We mainly collected the demographic information of patients, data at the initiation of ART, as well as outcome of follow-up. Demographic information including age, gender, marital status, route of HIV infection; data at the initiation of ART including WHO clinical stage, previous use of cotrimoxazole, initial antiretroviral regimen, baseline CD4^+^ cell count, ADE (if valid), and as well as other clinical data. All the collected information was used to the subsequent analyses.

### Definitions

AIDS-defining events: at least one of the diseases listed by the U.S. Centers for Disease Control (CDC) [30] or mentioned elsewhere. ART, a treatment regimen consisting of one or more of the following three drugs, including NRTIs, PIs and NNRTIs.

Endpoint: Death was validated as an endpoint, and censoring occurrence included lost to follow-up, transfer to another hospital, or cessation of ART.

### Statistical analysis

Chi square test was used to compare demographic characteristics and mortality rates between the ADE group and the non-ADE group. Kaplan–Meier analysis was used to calculate the accumulated mortality. We used the stepwise selection method to choose the variables to enter into the multi-variable analysis model, and the cox model was used to calculate mortality risk. Then, 1:1 propensity score matching (PSM) was used to select individuals to eliminate the effects of the characteristics that were statistically different between the ADE and the non-ADE groups. PSM used a calliper starting with 0.02, and all statistical variables matched with 0.0001 callipers at the end. The analyses and mapping were performed using GraphPad Prism version 5.0 (GraphPad Software, San Diego, California, USA), Origin 2018, or Statistical Package for the Social Sciences (SPSS) version 22.0 (SPSS Inc. Chicago, USA). The significance level was set at 0.05. All hypothesis tests were two-sided.

### Ethical considerations

All records were extracted from the Internet-based NFATP system. The study was approved by the Human Research Ethics Committee of Guangxi Medical University (Ethical Review No. 2019-SB-102). Every participant in this study voluntarily signed the written informed consent. We guarantee that ID number, name, address will be replaced by digital codes in the analysis dataset. In addition, the access of the data used for analysis has been limited inside the task forces and will not reveal to others. Individuals involved in this study maybe at risk of the release of their HIV status, revealing of their private information, and facing social stigma as well. However, participants will benefit from the ART program which can improve their prognosis, help to enjoy their normal life. The public health community can also benefit from this study by understanding the role of ART has played in the prevention of ADE.

## Results

### Baseline characteristics

**Fig. 1:**
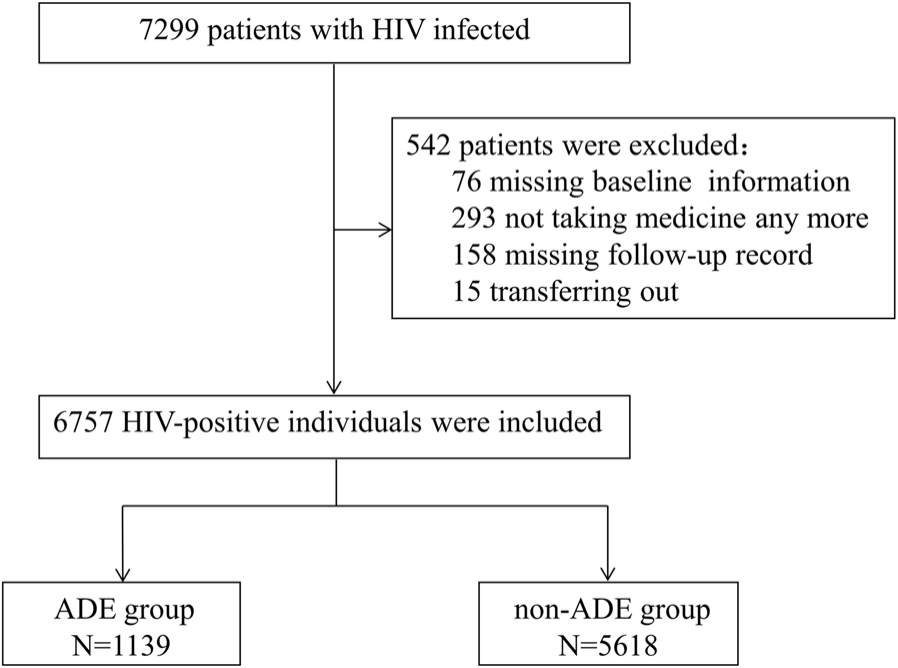
Flow charts of inclusion and exclusion criteria for research subjects in this study

The study included 7299 HIV/AIDS patients who initiated ART between January 2004 and December 2018 from the NFATP. Of those, 542 patients were excluded, including 76 without baseline information, 293 who quit ART, 158 without follow-up records, and 15 transferred to other hospitals. Ultimately, we enrolled 6757 eligible patients with 29,096.6 person-years of follow-up (Fig. 1). Among eligible patients, 1139 (16.86%) were with ADE (ADE group) and 5618 (83.14%) were without ADE (non-ADE group). The demographic characteristics of HIV/AIDS patients at ART initiation in the non-ADE and ADE groups were shown in Table 1. Between the two groups there was significant difference (*P* < 0.05) found for age, gender, route of HIV infection, WHO clinical stage, previous use of cotrimoxazole, and baseline CD4^+^ cell count (Table 1).

**Table 1:** Demographic characteristics of HIV/AIDS patients at ART initiation

Demographic characteristics	Total n (%)	ADE group n (%)	Non-ADE group n (%)	χ^2^	*P*
Total	6757	1139	5618		
Age				39.41	< 0.001
≤ 40	2108 (31.2)	279 (24.50)	1829 (32.56)		
40<age ≤ 65	3734 (55.26)	724 (63.56)	3010 (53.58)		
> 65	915 (13.54)	136 (11.94)	779 (13.87)		
Gender				48.23	< 0.001
Male	4619 (68.36)	878 (77.09)	3741 (66.59)		
Female	2138 (31.64)	261 (22.91)	1877 (33.41)		
Marital status				1.766	0.414
Unmarried	715 (10.58)	109 (9.57)	606 (10.79)		
Married or living with a partner	5026 (74.38)	851 (74.71)	4175 (74.31)		
Divorced or widowed or other	1016 (15.04)	179 (15.72)	837 (14.9)		
Route of HIV infection				11.094	0.004
Blood or plasma transfusion	352 (5.21)	45 (3.95)	307 (5.46)		
Sexual transmission	6104 (90.34)	1059 (92.98)	5045 (89.80)		
Other or unknown	301 (4.45)	35 (3.07)	266 (4.73)		
WHO clinical stage				1507.914	< 0.001
I	2518 (37.27)	74 (6.50)	2444 (43.50)		
II	1138 (16.84)	125 (10.97)	1013 (18.03)		
III	1867 (27.63)	290 (25.46)	1577 (28.07)		
IV	1234 (18.26	650 (57.07)	584 (10.40)		
Previous use of the cotrimoxazole				580.705	< 0.001
Yes	1879 (27.81)	649 (56.98)	1230 (21.89)		
No	4878 (72.19)	490 (43.02)	4388 (87.11)		
Initial antiretroviral regimen				0.61	0.737
2NRTIs + 1NNRTI	5943 (87.95)	1002 (87.97)	4941 (87.95)		
2NRTIs + 1PI	517 (7.65)	83 (7.29)	434 (7.73)		
Single or two drugs	297 (4.4)	54 (4.74)	243 (4.33)		
Baseline CD4^+^ cell count (cells/μL)				721.107	< 0.001
CD4 < 50	1900 (28.12)	675 (59.26)	1225 (21.80)		
50 ≤ CD4 < 100	796 (11.78)	137 (12.03)	659 (11.73)		
100 ≤ CD4 < 200	1430 (21.16)	169 (14.84)	1261 (22.45)		
200 ≤ CD4 < 350	1796 (26.57)	119 (10.45)	1677 (29.85)		
CD4 ≥ 350	835 (12.36)	39 (3.42)	796 (14.17)		
Duration of follow-up (years)^a^	4.00 (1.92–6.50)	3.58 (1.75–6.00)	4.17 (2.00–6.58)	3.64	< 0.001

*NRTI* nucleoside reverse transcriptase inhibitor, *NNRTI* non-nucleoside reverse transcriptase inhibitor means, *PI* protease inhibitor

^a^Data are presented as medium,  interquartile range (IQR), and T tests were used to compare the characteristics between the two groups

### The causes of death by ADE

Of all 6757 patients, 731 patients died, and 21.61% (158/731) died of ADE. Among the causes by ADE, the most common cause of death was disseminated mycosis (31.65%), followed by recurrent severe bacterial pneumonia (28.48%), herpes zoster (17.72%), extra-pulmonary tuberculosis (8.86%), and candidiasis of the oesophagus (5.70%) (Table 2).

**Table 2:** The causes of death by ADE

Death causes by ADE	Death number	Percentage of deaths (%)
Disseminated mycosis	50	31.65
Recurrent severe bacterial pneumonia	45	28.48
Herpes zoster	28	17.72
Extrapulmonary tuberculosis	14	8.86
Candidiasis of the esophagus	9	5.70
Pneumocystis pneumonia	4	2.53
Talaromyces marneffei infection	3	1.90
Disseminated non-tuberculous mycobacterium infection	2	1.27
Cryptococcal infection of the lung	2	1.27
Chronic herpes simplex virus infection	1	0.63
Total death number	158	100

### ADE group had higher mortality

**Fig. 2:**
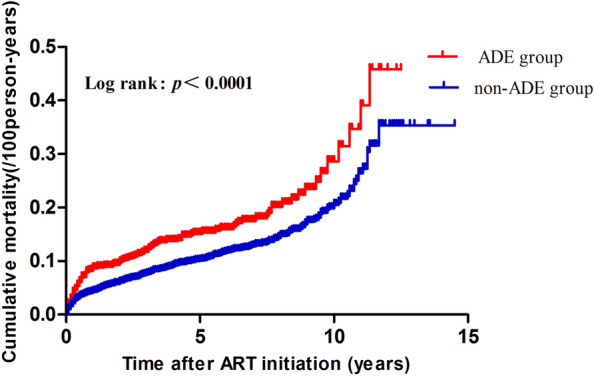
Kaplan-Meier analysis of cumulative mortality of HIV/AIDS patients, grouped by ADE (The log-rank test was used for statistical)

A total of 731 out of 6757 (10.81%) patients died during the 14-year period of follow-up, which contributed to a mortality rate of 2.51/100 person-years (95% CI 2.33–2.69); The mortality rates of the ADE group and the non-ADE group were 3.45/100 person-years (95% CI 2.92–3.97) and 2.34/100 person-years (95% CI 2.15–2.52), respectively; the mortality rate of the ADE group was significantly higher than that of the non-ADE group (Table 3). In addition, Fig. 2 showed that the ADE group had a higher cumulative mortality rate than the non-ADE group when ART lasted for 5 years or longer (log-rank: *P* < 0.0001) (Table 3).

**Table 3:** Comparison of mortality rate of HIV/AIDS patients between ADE and non-ADE group

Group	Total (n)	Deaths n (%)	*χ*^*2*^	P*	Person-years	Deaths/100 person-years (95% CI)	P**
ADE	1139	158 (13.87)	13.238	< 0.001	4586.17	3.45 (2.92–3.97)	< 0.0001
Non-ADE	5618	573 (10.20)			24,510.42	2.34 (2.15–2.52)	
Total	6757	731 (10.82)			29,096.6	2.51 (2.33–2.69)	

P* by Chi squared test; P** by log-rank test

### Factors related to mortality among HIV/AIDS patients on ART

The cox model was used to identify factors related to death. Univariate analysis showed that the patients with ADE had a higher mortality rate than those patients without ADE, with a hazard ratio (HR) of 1.618 (95% CI 1.356–1.931, *P *< 0.001). Multivariate analysis showed that the ADE group had a higher death risk than the non-ADE group, with an adjusted hazard ratio (aHR) of 1.291 (95% CI 1.061–1.571, *P *= 0.011). Cox analysis indicated that ADE, older age, male gender, previous non-use of cotrimoxazole, advanced WHO clinical stage, and low baseline CD4^+^ cell count were the risk factors for death (Table 4).

**Table 4:** Cox analysis of risk factors for the death of HIV/AIDS patients on ART

Variable	n	Univariate analysis	*P*	Multivariate analysis	*P*
ADE					
No	5618	1.00		1.00	
Yes	1139	1.618 (1.356–1.931)	< 0.001	1.291 (1.061–1.571)	0.011
Age			< 0.001		< 0.001
≤ 40	1210	1.00		1.00	
40 < age ≤ 65	3734	2 (1.664–2.404)	< 0.001	2.068 (1.699–2.518)	< 0.001
> 65	915	3.927 (3.191–4.831)	< 0.001	3.653 (2.896–4.607)	< 0.001
Gender					
Male	4619	1.00		1.00	
Female	2138	0.475 (0.394–0.574)	< 0.001	0.563 (0.463–0.686)	< 0.001
Marital status			0.027		0.069
Unmarried	715	1.00		1.00	
Married or living with a partner	5026	1.171 (0.893–1.536)	0.253	0.737 (0.551–0.985)	0.039
Divorced or widowed or other	1016	1.462 (1.071–1.994)	0.017	0.845 (0.601–1.187)	0.331
Route of HIV infection			0.372		
Blood or plasma transfusion	352	1.00			
Sexually transmitted	6104	1.205 (0.926–1.568)	0.166		
Other or unknown	301	1.139 (0.757–1.714)	0.533		
WHO clinical stage			< 0.001		< 0.001
I	2510	1.00		1.00	
II	1132	0.954 (0.733–1.242)	0.727	0.863 (0.7061.055)	0.150
III	1863	1.057 (0.847–1.319)	0.625	1.095 (0.838–1.430)	0.506
IV	1218	1.624 (1.281–2.058)	< 0.001	1.454 (1.092–1.935)	0.01
Previous use of the cotrimoxazole					
Yes	1879	1.00		1.00	
No	4878	0.406 (0.34–0.486)	< 0.001	0.4880.403–0.592	< 0.001
Initial antiretroviral regimen			< 0.001		< 0.001
2NRTIs + 1NNRTI	5943	1.00		1.00	
2NRTIs + 1PI	517	6.212 (4.762–8.103)	< 0.001	4.721 (3.577–6.23)	< 0.001
Single or two drugs	297	1.122 (0.844–1.49)	0.428	1.163 (0.87–1.555)	0.307
Baseline CD4^+^ cell count (cells/μL)			< 0.001		< 0.001
CD4 ≥ 350	835	1.00		1.00	
200 ≤ CD4 < 350	1796	0.742 (0.442–1.247)	0.260	0.794 (0.468–1.345)	0.39
100 ≤ CD4 < 200	1430	1.263 (0.762–2.095)	0.365	1.244 (0.739–2.094)	0.41
50 ≤ CD4 < 100	796	1.484 (0.886–2.487)	0.134	1.56 (0.911–2.669)	0.11
CD4 < 50	1900	1.898 (1.162–3.103)	0.011	1.846 (1.095–3.114)	0.02

### Propensity score matching (PSM) analysis

As Table 1 has shown, demographic characteristic variables including age, gender, route of HIV infection, WHO clinical stage, previous use of cotrimoxazole, and baseline CD4^+^ cell counts were statistically different between the two groups. PSM analysis was used to match those characteristic variables between the two groups. Totally, 1720 patients (860 ADE and 860 non-ADE patients) were included. Chi squared test showed the differences in the above variables were not significant between the two groups after matching (Additional file 1: Table S1). The adjusted cox analysis showed that the ADE group still had a higher mortality rate than the non-ADE group (HR = 1.325, 95% CI 1.016–1.728, *P *= 0.038; aHR = 1.581, 95% CI 1.192–2.099, *P *= 0.002) (Table 5).

**Table 5:** Crude and adjusted risk ratios for predictors of mortality in ADE and non-ADE groups

ADE	Total patients, n	Deaths n (%)	Deaths/100 person-years (95% CI)	HR (95% CI)	*P*	aHR (95% CI)	*P*
No	860	100 (11.63)	2.80 (2.26–3.33)	1.00	-	1.00	-
Yes	860	124 (14.42)	3.41 (2.83–4.00)	1.325 (1.016–1.728)	0.038	1.581 (1.192–2.099)	0.002

*aHR* adjusted by age at ART, gender, marital status, route of HIV infection, WHO clinical stage before ART, Previous use of the cotrimoxazole, initial antiretroviral regimen, baseline CD4^+^ cell count

## Discussion

In this study, the prevalence of ADE in HIV/AIDS patients at ART initiation was 16.86%, which was roughly consistent with the data of previous studies in other countries or regions; for example, a report from France showed that 16% of patients with newly diagnosed HIV were ADE [31]. Our study also showed that even when individuals were on ART, the mortality of patients with ADE was significantly higher than that of those without ADE, which was consistent with some previous studies: a large multi-centre cohort study showed that non-AIDS mortality was twice as high among patients with an ADE compared to without an ADE [32], and this multiple is a little higher than that of this study; a research showed that HIV controllers experience non-ADE, albeit at lower rates than patients who do not spontaneously control the virus [33]. While in this study, disseminated mycosis, recurrent severe bacterial pneumonia, herpes zoster, extra-pulmonary tuberculosis, and candidiasis of the oesophagus were the top causes of death by ADE.

Previous studies have shown that patients with a late diagnosis of HIV infection had a higher risk of mortality than those with an early HIV diagnosis [34], and universal ART among late presentation patients reduced mortality by just 10% [35]. Late presentation is defined well as, a patient diagnosed with the first CD4^+^ cell count < 350/μL, or a patient with AIDS-defining illness regardless of CD4^+^ cell count during diagnosis [8, 36, 37]. In this study, the median CD4^+^ cell count in the ADE group was 29.00 cells/μL (IQR: 12.00–116.50), and 96.05% of patients with ADE had late presentation, demonstrating that most of patients with ADE were in an advanced stage of HIV/AIDS. And studies showed that low CD4^+^ cell count (baseline CD4 count ≤ 50 cells/μL) was related to treatment failure [38, 39], a lower CD4^+^ cell count was always associated with a higher risk of a new AIDS events or death [40].

A delay in the diagnosis of patients with ADE postpones HIV care and the administration of ART, which further reduces the possibility of achieving viral suppression. In addition, even on ART, most of the patients with ADE at advanced HIV/AIDS stages may also have failed immunological recovery, thus increasing the risk of morbidity and mortality.

A number of studies have shown that early diagnosis and treatment at a higher CD4^+^ cell count without ADE could benefit patients in terms of preserving immune function and reducing the risk of mortality [20, 41, 42]. However, many HIV-infected people were unaware of their infection status. In the United States, 15% of HIV/AIDS patients were unaware of their HIV infection status [43], and only 40% of the adult population had undergone HIV testing [44]. HIV screening, especially in HIV high risk populations including men who have sex with men (MSM), female sexual workers (FSW), and bisexuals may be valuable for earlier diagnosis [45]. Therefore, to reduce HIV transmission and improve the effect of ART, HIV infection needs to be diagnosed as early as possible. Our study found that patients aged from 40 to 65 years, following sexual transmission, with previous non-use of cotrimoxazole, and a low baseline CD4^+^ cell count tended to have a higher risk of death. Therefore, increasing active HIV testing coverage and frequency should be implemented in high-risk populations. Once HIV infection is confirmed, ART should be provided immediately, which is expected to reduce the incidence of ADE and thus reduce mortality.

The study had a large sample size and long-term investigation, so that the study has a certain representative of ART outcomes in Guangxi and Southwestern China. In addition, this data collection began in 2004, and the cohort was well followed-up through the whole NFATP period in China, which can fully evaluate the impact of ADE on mortality for patients on ART. Several limitations of our study should also be mentioned. Firstly, this study is a retrospective cohort study, and the subjects were from a city in Guangxi, which may lead to a loss of access bias and selection bias. However, the large sample size (HIV/AIDS patients may come from other places in Guangxi or the country) may reduce these biases to some extent. Secondly, many patients in this study had no viral load data at the initiation of ART, and we could not assess the impact of viral load on treatment outcomes. Thirdly, our analysis did not include surveys of patient compliance with medication.

In conclusion, in the era of ART, it is still common for patients with initial ART to have ADE. Even on ART, these patients have a higher risk of death than those without ADE. Currently there is no cure for HIV/AIDS, and we should further promote voluntary testing and improve people’s awareness of early diagnosis to reduce late presentations, especially those with ADE.

## Supplementary information

**Additional file 1: Table S1.** After PSM, demographic characteristics of HIV/AIDS patients at initiation of ART.
