# Supplementary material for: Effect of AIDS-defining events at initiation of antiretroviral therapy on long-term mortality of HIV/AIDS patients in Southwestern China: a retrospective cohort study
Source: AIDS Res Ther. 2020 Jul 17;17:44. doi: 10.1186/s12981-020-00300-4 (PMC7367222; doi:10.1186/s12981-020-00300-4)
Supplement: Supplementary file 1 — Additional file 1: Table S1. After PSM, demographic characteristics of HIV/AIDS patients at initiation of ART. [file 12981_2020_300_MOESM1_ESM.docx]

**Effect of** **AIDS-defining events** **at initiation of antiretroviral therapy** **on long-term mortality of HIV/AIDS patients in southwestern China: a retrospective cohort study**

Yunxuan Huang^1#^, Oulu Zhou^2,3#^, Zhigang Zheng^3^, Yuexiang Xu^1^, Yi Shao^4^, Chunwei Qin^1^, Fengxiang Qin^3^, Jingzhen Lai^3^, Huifang Liu^3^, Rongfeng Chen^2,3^, Li Ye^3^, Hao Liang^2,3^, Xionglin Qin^1*^, Junjun Jiang^3^

| **Table S1. After PSM, demographic characteristics of HIV/AIDS patients**  **at initiation of ART** | | | | | |
| --- | --- | --- | --- | --- | --- |
| **Demographic characteristics** | **Total**  **n(%)** | **ADE group**  **n(%)** | **non-ADE group**  **n(%)** | **χ2** | ***P*** |
| **Age** |  |  |  | 0.357 | 0.837 |
| ≤40 | 412(23.95) | 201(23.37) | 211(24.53) |  |  |
| 40＜age≤65 | 1112(64.65) | 559(65.00) | 553(64.30) |  |  |
| ＞65 | 196(11.40) | 100(11.63) | 96(11.16) |  |  |
| **Gender** |  |  |  | 0.401 | 0.527 |
| Male | 1329(77.27) | 670(77.91) | 659(76.63) |  |  |
| Female | 391(22.73) | 190(22.09) | 201(23.37) |  |  |
| **Marital status** |  |  |  | 2.36 | 0.307 |
| Unmarried | 151(8.78) | 81(9.42) | 70(8.14) |  |  |
| Married or living with a partner | 1330(77.33) | 669(77.79) | 661(76.86) |  |  |
| Divorced or widowed or other | 239(13.90) | 110(12.79) | 129(15.00) |  |  |
| **Route of HIV infection** |  |  |  | 0.098 | 0.952 |
| Blood or plasma transfusion | 71(4.13) | 36(4.19) | 35(4.07) |  |  |
| Sexually transmitted | 1601(93.08) | 801(93.14) | 800(93.02) |  |  |
| Other or unknown | 48(2.79) | 23(2.67) | 25(2.91) |  |  |
| **WHO clinical stage** |  |  |  | 0.622 | 0.891 |
| I | 140(8.14) | 72(8.37) | 68(7.91) |  |  |
| II | 241(14.01) | 116(13.49) | 125(14.53) |  |  |
| III | 543(31.57) | 269(31.28) | 274(31.86) |  |  |
| IV | 796(46.28) | 403(46.86) | 393(45.7) |  |  |
| **Previous use of the cotrimoxazole** |  |  |  | 1.34 | 0.247 |
| Yes | 852(49.53) | 414(48.14) | 438(50.93) |  |  |
| No | 868(50.47) | 446(51.86) | 422(49.07) |  |  |
| **Initial antiretroviral regimen** |  |  |  | 2.37 | 0.306 |
| 2NRTIs+1NNRTI | 1560(90.70) | 773(89.88) | 787(91.51) |  |  |
| 2NRTIs+1PI | 101(5.87) | 58(6.74) | 43(5.00) |  |  |
| Single or two drugs | 59(3.43) | 29(3.37) | 30(3.49) |  |  |
| **Baseline CD4^+^ cell count (cells/ μL)** |  |  |  | 1.979 | 0.74 |
| CD4＜50 | 935(54.36) | 472(54.88) | 463(53.84) |  |  |
| 50≤CD4＜100 | 228(13.26) | 109(12.67) | 119(13.84) |  |  |
| 100≤CD4＜200 | 276(16.05) | 144(16.74) | 132(15.35) |  |  |
| 200≤CD4＜350 | 209(12.15) | 103(11.98) | 106(12.33) |  |  |
| CD4≥350 | 72(4.19) | 32(3.72) | 40(4.65) |  |  |
